# Incidence of spine surgery for degenerative and traumatic pathologies in patients with a history of cancer: a nationwide register-based study between 1997 and 2020 from Finland

**DOI:** 10.2340/17453674.2025.44247

**Published:** 2025-07-07

**Authors:** Leevi A TOIVONEN, Ville PONKILAINEN, Jussi P REPO, Ville M MATTILA

**Affiliations:** Department of Orthopedics and Traumatology, Tampere University Hospital and Tampere University, Tampere, Finland

## Abstract

**Background and purpose:**

The number of cancer survivors has increased. Although spine surgery rates have multiplied in the general population, they are understudied in cancer populations. We aimed to determine the incidence rates of spinal surgery for degenerative and traumatic pathologies in patients with prior cancer. Our secondary aim was to define the underlying primary cancer diagnoses and survival rates after spinal procedures.

**Methods:**

Data was combined from 3 nationwide registers: the Finnish Cancer Register, Finnish Care Register for Health Care, and Finnish Cause of Death Register. Spine surgeries were identified using diagnosis and procedural codes, and tumor surgeries were excluded. Incidence rates were calculated per 100,000 inhabitants and adjusted for age and sex. Kaplan–Meier survival estimates (with 95% confidence intervals [CI]) were calculated per the first spine surgery.

**Results:**

10,280 patients underwent 12,425 surgeries, with a mean age of 70 years; 53% were women. Degenerative pathologies accounted for 74% of the surgeries, followed by disc pathologies (20%) and trauma (6%). The incidence of spine surgeries increased from 3.7 to 15.1 per 100,000 person-years (300%) between 1997 and 2019. The increase mostly occurred in degenerative spine procedures (420%), whereas disc and trauma surgeries were temporally stable. The most common previously diagnosed cancers were breast (24%) and prostate (22%) cancers. All-cause survival after spine surgery was 94% (CI 94–95) at 1 year, and cancer-specific survival was 90% (CI 0.89–0.91) at 15 years.

**Conclusion:**

We showed a 300% increase in spine surgeries unrelated to cancer in patients with a history of cancer between 1997 and 2020. Survival rates remained favorable (94% [CI 0.89–0.91] at 1 year).

As cancer rates continue to increase, advancements in diagnostics and care have improved survival [1,2]. With many cancers, such as lymphoma, melanoma, breast, prostate, thyroid, endometrial, and testicular cancer, survival rates of over 80% have been reported at 5 years [2]. As a result, the number of patients with stable or slowly progressing or cured cancer continues to grow. The long-term quality of life of patients with prior cancer is a subject of increasing interest in research [3]. We expect their functional demands to rise along with increasing survival.

In the general population, increasing rates of spinal surgery have been reported worldwide [4-6]. Degenerative spine disease, predominantly manifesting as some form of spinal stenosis, is the major cause of elective spinal surgery. In Finland, decompression procedures are increasingly outnumbering fusion surgeries [5] whereas disc herniation surgery is decreasing [7]. The reported increase in spine fracture surgeries in Finland is mostly seen in older individuals and for cervical spine fractures [8]. Metastatic spine disease is common in patients with advanced cancer, and it can be the first manifestation of cancer progression or undiagnosed cancer [9-11]. Data on spine surgeries unrelated to spinal metastases in prior cancer populations is not available, although we expect these surgeries increasingly to burden healthcare systems.

The objective of this nationwide registry-based study was to determine the incidence rates of spinal surgery for degenerative and traumatic pathologies in patients with prior cancer. The secondary aim was to define the underlying primary cancer diagnoses and survival rates after spinal procedures.

## Methods

### Data sources and linking

This retrospective study was based on data retrieved from 3 nationwide registries: the Finnish Cancer Registry (FCR), the Finnish Care Register for Healthcare (FCRH), and the Finnish Cause of Death Register. The FCR has collected population-based data on cancer incidence since 1953 with excellent coverage and validity for solid tumors benchmarked against hospital inpatient data [12]. The FCRH contains hospital inpatient data with excellent coverage and accuracy, validated against several external databases [13-15]. The Finnish Cause of Death Register covers all registered deaths of Finnish inhabitants. All Finnish inhabitants have their own personal identification number allowing unambiguous linkage across national registries.

### Sample formation

All patients in FCR between 1964 and 2021 were screened for spine surgery-related hospitalization between 1997 and 2020. The spine-surgery-related procedural codes used in the screening are listed in Table S1 (see Supplementary data). To be included in the study, the potential candidates had to have an FCR record including a cancerous code (non-melanotic skin cancers [C44] and benign tumors [D*] as the primary diagnosis at the spine-surgery-related hospitalization were excluded) prior to spine surgery-related hospitalization. In addition, patients were excluded if they had a cancer-related primary diagnosis, or tumor resection as a procedural code in spine-surgery-related hospitalization (Table S1, see Supplementary data).

Patients were classified based on their indication for spine surgery as follows: (i) degenerative pathologies (predominantly spinal stenosis, spondylolisthesis, and spondylolysis), (ii) disc pathologies (disc herniations), and (iii) traumas (vertebral fractures). Patients with tumors and pathological fractures were excluded. Spondylolysis was combined with the degenerative cohort, as it mostly was coded as “M43.1 spondylolisthesis,” which could not be differentiated from degenerative spondylolisthesis. Furthermore, in the study sample where only a small minority were aged below 40 years, spondylolysis led to surgery practically only in cases with advanced segmental degeneration caudal to the pars defect.

### Statistics

Continuous variables were summarized using the median and interquartile range (IQR) for non-normally distributed data, whereas the mean and standard deviation (SD) were reported for normally distributed variables. Incidence rates were derived from annual mid-year population estimates obtained from the National Population Register (Official Statistics of Finland). Incidence rates were expressed per 100,000 individuals and adjusted for age based on the 10-year age and sex distributions of the Finnish population. Kaplan–Meier (KM) analysis was used to estimate survival probabilities following surgery, providing 95% confidence intervals (CI). The follow-up period extended until December 30, 2020, with censoring occurring at that point, while deaths before this date were considered events. The incidence rates reflected the number of procedures performed, whereas survival analyses were based on each patient’s first spinal surgery after cancer diagnosis.

To further explore variations in incidence, subgroup analyses were performed based on age, surgical approach (fusion or non-fusion), and primary malignancy. Due to the relatively small sample size, incidence rates were smoothed using 3-year rolling averages when the rates were grouped into more than 3 groups (age and primary cancer). Changes in incidence were reported as relative percentage changes. Because the dataset contained no missing values, the imputation techniques were unnecessary. All statistical analyses were conducted using R version 4.3.1 (R Foundation for Statistical Computing, Vienna, Austria). The study adhered to STROBE guidelines [16].

### Ethics, data sharing, funding, and conflicts of interest

The Finnish Health and Social Data Permit Authority (FinData) authorized the study (THL/3527/14.02.00/2023), collected and combined data from the registries, and handled data to the authors in a pseudonymized form. The authors do not have access to the pseudonymization key. The data supporting the findings of this study are available from FinData with permission; however, the authors do not have the authority to share the data. This study received funding from State funding for university-level health research, Tampere University Hospital, Wellbeing Services County of Pirkanmaa. The authors declare no conflicts of interest. Complete disclosure of interest forms according to ICMJE are available on the article page, doi: 10.2340/17453674.2025.44247

## Results

**Figure 1:**
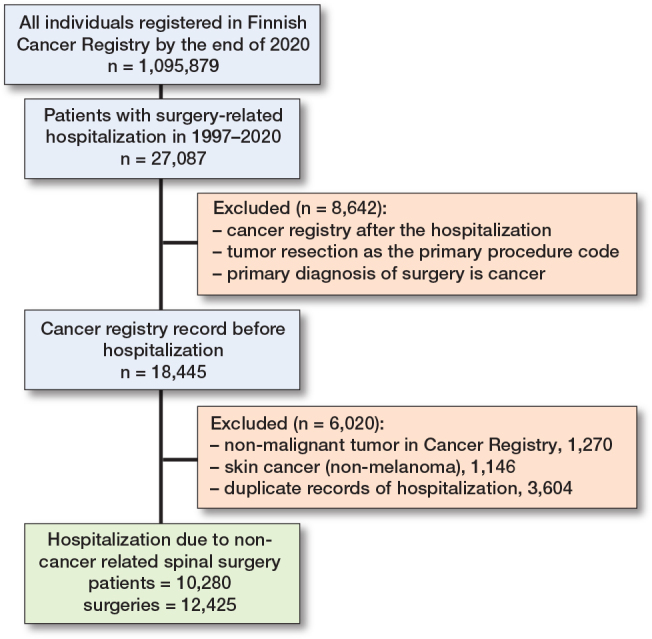
Flowchart showing patient inclusion.

The data included 10, 280 patients with a history of cancer who underwent 12,425 spine surgeries unrelated to cancer (Figure 1). The mean (IQR) age of the patients at the time of the first surgery was 70.1 years (61.6–76.7), and 52.8% of the patients were female (Table 1). Most procedures were performed for degenerative pathologies (n = 9,228, 74%), while surgery was less common for disc pathologies (n = 2,490, 20%) and trauma (n = 707, 6%).

**Table 1:** Patient characteristics and the proportion of fusion procedures of all surgeries according to main surgical cohorts

Item	n	Age mean (IQR)	Women n (%)	Fusion n (%)
All	12,425	70 (62–77)	6,564 (53)	3,840 (31)
Degenerative	9,228	72 (64–78)	4,986 (54)	2,843 (31)
Disc pathologies	2,490	61 (52–70)	1,304 (52)	415 (17)
Trauma	707	73 (64–79)	274 (39)	582 (82)

IQR = interquartile range.

### Incidence rates

**Figure 2:**
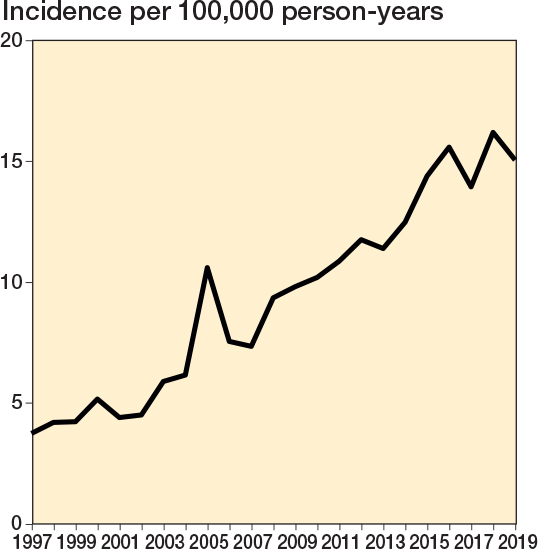
Annual age- and sex-adjusted incidence of spine surgery among patients with previous cancer from 1997 to 2019 in Finland.

**Figure 3:**
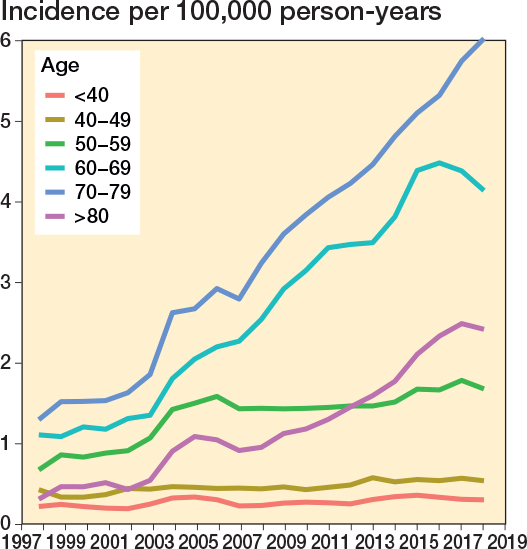
Annual age- and sex-adjusted 3-year rolling mean of incidence of spine surgery separated by age among patients with previous cancer from 1997 to 2019 in Finland.

**Figure 4:**
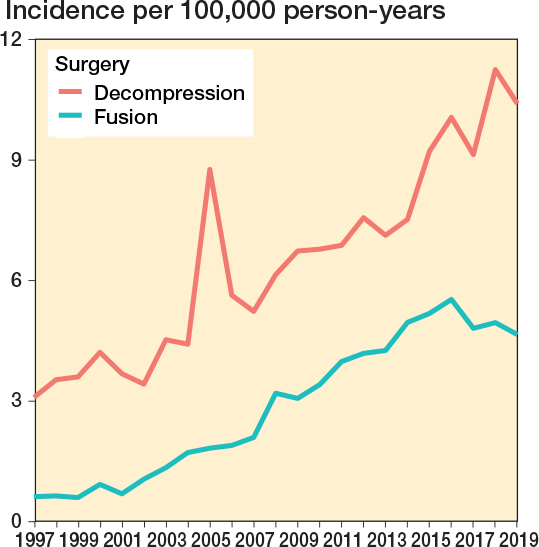
Annual age- and sex-adjusted incidence of spine surgery separated by decompression vs fusion among patients with previous cancer from 1997 to 2019 in Finland.

**Figure 5:**
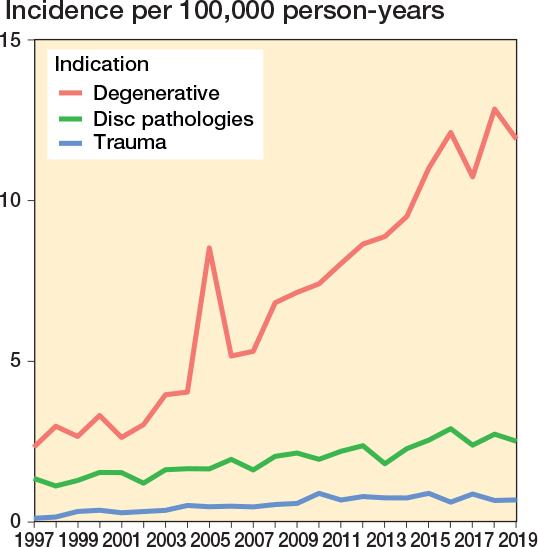
Annual age- and sex-adjusted incidence of spine surgery separated by pathology type among patients with previous cancer from 1997 to 2019 in Finland.

The annual age- and sex-adjusted incidence rate of spine surgery in patients with a history of cancer increased considerably from 3.7 in 1997 to 15.1 in 2019 (300% increase) (Figure 2). The increase was greatest in patients aged 70–79 years at surgery (3-year rolling mean from 1.3 in 1998 to 6.0 per 100,000 person-years in 2018, a 360% increase) (Figure 3). The surgery rate remained consistently low in the youngest age groups (< 50 years). The increase in surgery rates almost completely occurred in surgeries for degenerative pathologies (from 2.3 to 12 per 100,000, 420% between 1997 and 2019), whereas surgery rates for disc pathologies and fractures remained consistent (Figure 4). Most of the procedures were decompressions (n = 8,585, 69%) (see Table 1). Both decompression and fusion procedures showed a marked increase over the study period, rising from 3.1 to 10.1 per 100,000 person-years (225% increase) and from 0.64 to 4.9 per 100,000 person-years (666% increase), respectively (Figure 5).

**Figure 6:**
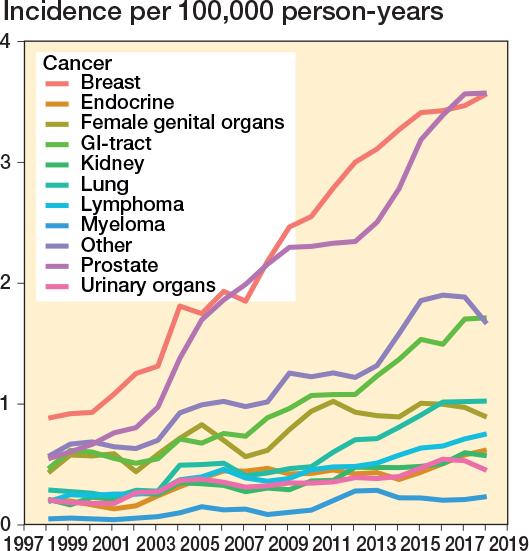
Annual age- and sex-adjusted 3-year rolling mean of incidence of spine surgery separated by cancer diagnosis among patients with previous cancer from 1997 to 2019 in Finland.

**Figure 7:**
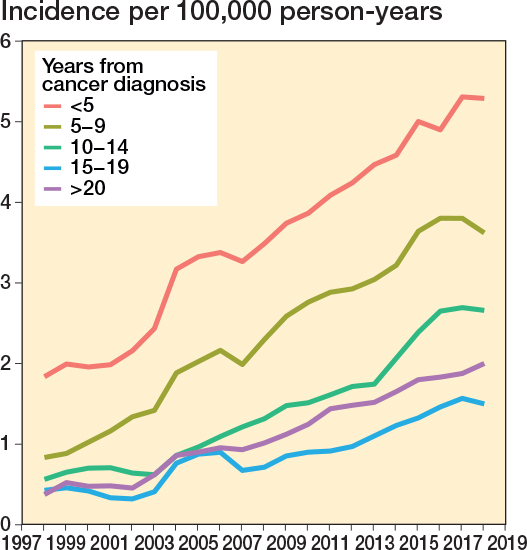
Annual age- and sex-adjusted 3-year rolling mean of incidence of spine surgery separated by interval between previous cancer diagnosis and spine surgery from 1997 to 2019 in Finland.

The most prevalent specific cancer diagnoses during the study period were breast cancer (n = 2,513, 24%), prostate cancer (n = 2,232, 22%), GI tract cancer (n = 1,075, 11%), cancer of the female genital organs (n = 826, 8%), lung cancer (n = 641, 6%), lymphoma (n = 478, 5%), cancer of endocrine organs (n = 393, 4%), kidney cancer (n = 392, 4%), cancer of urinary organs (n = 372, 4%), and myeloma (n = 157, 2%) (Table 3). Breast and prostate cancers were associated with an increased rate of subsequent, non-cancer-related spinal surgery (Figure 6). While most spinal surgeries occurred within a few years after cancer diagnosis, the proportion of those with a longer interval increased over time (Figure 7).

### Survival analysis

For the entire study period, the all-cause survival probabilities for the entire cohort were 94% (CI 94–95) at 1 year, 91% (CI 90–91) at 2 years, 81% (CI 80–82) at 5 years, and 70% (CI 70–71) at 10 years (Table 2). The survival probabilities according to the previous cancer diagnoses were highest in breast cancer (97% [CI 96–97]) and cancer of endocrine organs (97% [CI 96–99]) at 1 year (Table 3).

**Table 2:** Kaplan–Meier estimates of all-cause survival after non-cancer-related spine surgery among patients with cancer history from 1997 to 2020 in Finland (N = 10,280)

Follow-up^a^	Number at risk	Survival probability (CI), %
1-year	9,708	94 (94–95)
2-year	9,339	91 (90–91)
5-year	8,342	81 (80–82)
10-year	7,238	70 (70–71)

a Median (IQR) follow-up was 5.4 (2.5–9.9) years.

CI = 95% confidence interval; IQR = interquartile range.

**Table 3:** Survival probabilities of patients undergoing non-cancer-related spine surgery between 1997 and 2020 in Finland. Survival probabilities are categorized by primary cancer diagnosis and presented as 1-, 2-, 5-, and 10-year probabilities

Primary diagnosis	n	1 year		2 years		5 -years		10 years		Follow-up, years Median (IQR)
		Number at risk	Survival probability % (CI)	Number at risk	Survival probability % (CI)	Number at risk	Survival probability % (CI)	Number at risk	Survival probability % (CI)	
Breast	2,513	2,270	97 (96–97)	2,040	94 (93–95)	1,458	85 (83–86)	700	69 (66–71)	6.1 (2.7–10.7)
Prostate	2,232	1,955	94 (93–95)	1,719	90 (88–91)	1,062	74 (72–76)	407	48 (45–51)	4.8 (2.2–8.5)
Other	1201	1,049	91 (90–93)	948	86 (84–88)	624	72 (70–75)	298	55 (51–58)	5.2 (2.3–9.9)
GI tract	1075	954	93 (92–95)	846	88 (86–90)	556	75 (72–78)	267	62 (58–66)	5.3 (2.3–9.6)
Female genital	826	748	96 (94–97)	690	93 (91–94)	510	80 (78–83)	247	54 (50–58)	6.7 (3.2–11.2)
Lung	641	641	96 (94–98)	508	93 (91–95)	346	84 (81–88)	161	69 (65–74)	5.4 (2.4–10.1)
Lymphoma	478	419	93 (91–95)	371	89 (86–92)	255	77 (73–81)	145	79 (74–84)	5.4 (2.2–10.1)
Endocrine	393	346	97 (96–99)	317	96 (94–98)	240	92 (89–95)	125	62 (57–67)	7.0 (2.7–13.0)
Kidney	392	342	93 (90–96)	306	88 (84–91)	204	74 (70–79)	94	54 (49–61)	5.4 (2.4–9.8)
Urinary organs	372	331	92 (90–95)	306	88 (85–92)	201	73 (68–78)	90	50 (44–56)	5.4 (2.8–9.8)
Myeloma	157	134	88 (83–93)	102	73 (67–81)	48	43 (36–53)	12	20 (13–30)	3.3 (1.5–5.9)

CI = 95% confidence interval; IQR = interquartile range.

**Figure 8:**
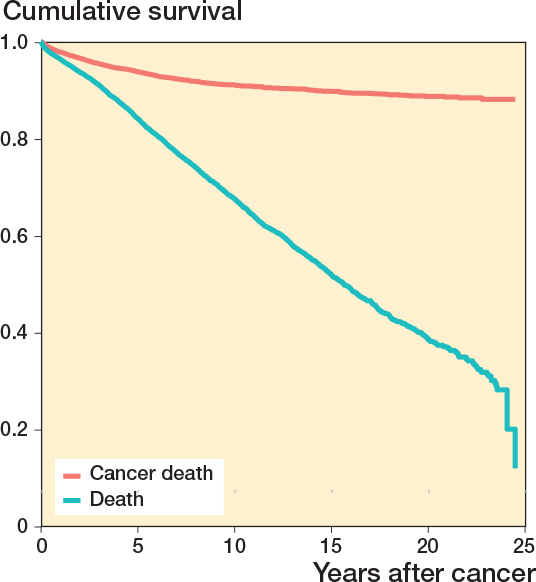
Kaplan–Meier curve of competing risk analysis of all patients undergoing non-cancer-related spine surgery after previous cancer from 1997 to 2020 in Finland.

Based on the competing risk analysis, cancer-specific survival at the 15-year time point was higher (90% [CI 0.89–0.91]) than all-cause survival (52% [CI 0.50–0.53]) (Table 4, Figure 8).

**Table 4:** Competing risk analysis of all patients who underwent primary spinal cancer surgery in Finland from 1997 to 2020

Follow-up	Number at risk	Cancer death		All-cause death	
		Number of deaths	Survival probability % (CI)	Number of deaths	Survival probability % (CI)
1-year	9,114	210	98 (98–98)	360	96 (96–97)
5-year	5,504	345	94 (93–94)	1,019	84 (83–85)
10-year	2,546	165	91 (90–92)	941	68 (66–69)
15-year	1,007	43	90 (89–91)	530	52 (50–53)

CI = 95% confidence interval.

## Discussion

This is the first study to report nationwide general spine surgery rates in a population with a prior cancer. We aimed to determine the incidence of non-cancer-related spine surgery in patients with a history of cancer and survival thereafter. We found a considerable increase in the rate of non-cancer-related spinal surgery in Finland in patients with previous cancer between 1997 and 2020. Surgery for degenerative spine conditions accounts for the observed increase in spinal procedures. Breast and prostate cancers were the most common diagnoses in patients with prior cancer undergoing subsequent spine surgery.

The observed increase in spine surgery for degenerative causes among patients with previous cancer (300% between 1997 and 2020) did not fall short of what Ponkilainen et al. reported in the general Finnish population (133% increase in lumbar decompression surgery from 1997 to 2018) [5]. They reported the steepest increase in lumbar fusion surgery among women aged > 75 years (422%) and still a notable increase among women aged from 55 to 75 years (289%). In the United States, fusion surgery increased by 139% from 2004 to 2015 among patients aged > 65 years [4]. In this study, surgery was focalized in the same age groups among patients with prior cancer. In the Finnish population, disc herniation surgery was reportedly most common at ages between 35 and 64 years, where the surgery rate decreased between 1997 and 2018, while it remained constantly low in older age groups [7]. This was consistent with our results in patients with previous cancer. Surgery for spinal trauma was remarkably low in this study. Previously, surgery rates for spine fractures have been reported to be increased in Finland among age groups > 60 years [8]. Considering the high risk of osteoporosis in patients with chronic cancer [17,18], the stable rate of spinal fracture surgery was surprising. As fracture surgeries were identified based on the same diagnosis codes as previously in the general population [8], we assume this finding is real. It may be explained by nonoperative management in some cases.

Breast and prostate cancers are the most prevalent cancers, accounting for almost half of the cancer prevalence in the population [19]. Some frequently occurring cancers, such as lung cancer and hematological cancers, generally have poorer prognoses, thus lowering their relative prevalence. In the Finnish female population, the next most prevalent cancers beyond breast cancer, namely colorectal cancer, corpus uteri cancer, and melanoma, are less than one-fifth in prevalence as compared with breast cancer [19]. In men, the next most prevalent cancers beyond prostate cancer are colorectal cancer, melanoma, and urinary tract cancer, with a prevalence of less than one-fourth that of prostate cancers [19]. This explains the preponderance of breast and prostate cancers in our data, although it was less pronounced than in the general population. Italian population-based data indicated higher cure rates for colorectal cancer (87% in women, 83% in men) and corpus uteri cancer (93%) than for breast cancer (72%) or prostate cancer (64%) [20]. Cured patients may more likely be deemed fit for surgery than patients with chronic cancer, which may have increased the surgery rates in those with prior colorectal cancer and corpus uteri cancer.

In our study, mortality rates exceeded those reported in the general spine surgery populations. Toivonen et al. reported a 5-year mortality rate of 3.4% (CI 2.2–5.4) in a lumbar fusion cohort and 4.8% (CI 3.5–6.7) in the age-, sex-, and comorbidity-adjusted general population cohort [21], which was lower than the 5-year mortality rate of 19% (CI 18–20). A review by Yavin et al. found no excess mortality secondary to lumbar spine procedures [22]. In contrast, a positive cancer history, even after curative treatment, is associated with excess mortality [18]. Cancer survivors experience a higher incidence of chronic comorbidities, frailty, and premature aging [18]. Whether this translates into accelerated spinal degeneration remains understudied.

Our data did not include the cure rate after cancer diagnosis, but the increasing interval from cancer diagnosis to subsequent spine surgery and high postoperative survival rates implied that many of our subjects may be cancer survivors. In fact, the competing risk analysis revealed that most patients died of causes other than their previously diagnosed cancer.

As the population of cancer survivors continues to grow, healthcare systems are increasingly facing their demands. The possibility of metastatic spine disease deserves continued attention in all practitioners. Besides this, degenerative spine pathologies place a burden on these populations. It is possible that less invasive methods and improved perioperative care have made surgery a viable option for the growing frail population, which may explain our observed 300% increase in surgeries.

### Limitations

First, the retrospective, register-based setting limited our analysis to what could be retrieved from the registers, for example, preventing insights into the outcomes of cancer treatment and clinical manifestations behind spine surgery. Lack of comprehensive comorbidity data precluded taking into account the subject’s frailty status, which definitely influences survival. Second, potential miscoding in the register data may have distorted our findings, but the generally high coverage and accuracy of the registries used likely limited this bias. Third, our data included only Finnish inhabitants, and data concerning emigration was missing, which may have marginally overestimated survival and underestimated the incidence of surgery. Fourth, as there are no established criteria for spinal surgery, the observed rates of surgery reflect local practices during the study period. An increasing incidence of spine surgery and cancer prevalence have been reported worldwide, indicating similar trends are present elsewhere as well.

### Conclusions

The number of surgeries for degenerative spine pathologies increased 300% whereas surgery rates for disc pathologies and fractures remained consistent between 1997 and 2020 among patients with a history of cancer in Finland. Postoperative all-cause survival was favorable at 94% (CI 94–95) at 1 year. Cancer-specific survival was 90% (CI 0.89–0.91) at 15 years whereas all-cause survival at that time was 52% (CI 0.50–0.53).

This data presents spine surgery as a viable option for the growing population of cancer survivors.

### Supplementary data

Table S1 including the NOMESCO procedural codes used to collect spinal surgeries is available as Supplementary data on the article page, doi: 10.2340/17453674.2025.44247

## Notes

LT planned the study and drafted the manuscript. VP performed the statistical analyses. All authors revised the manuscript and accepted its final version.

Handling co-editors: Paul Gerdhem and Robin Christensen

*Acta* thanks Yohan Robinson for help with peer review of this manuscript.
